# Cool mama: Temperature regulation during high-intensity interval running in pregnant elite and recreational athletes

**DOI:** 10.1016/j.jesf.2024.09.003

**Published:** 2024-09-14

**Authors:** Sofia Brevik-Persson, Christina Gjestvang, Emilie Mass Dalhaug, Birgitte Sanda, Jørgen Melau, Lene A.H. Haakstad

**Affiliations:** a Department of Sports Medicine, Norwegian School of Sports Sciences, Norway; b Joint Medical Service, Norwegian Armed Forces, Norway; c Arendal Gynekologi AS, Norway

**Keywords:** Athletes, High-intensity exercise, Temperature, Thermoregulation, Pregnancy, Running

## Abstract

**Background:**

Regular exercise during pregnancy is beneficial, but athletes often exceed the recommended 150 min of moderate-intensity activity, incorporate high-intensity exercises. The upper limit for exercise intensity and duration on fetal and maternal safety remains uncertain. A concern is a maternal core body temperature of >39.0 °C, potentially increase the risk of heat-related fetal malformations and complications during pregnancy. Blood flow redirection for thermoregulation could compromise fetal cardiovascular function, increasing the risk of miscarriage and preterm labor. This study evaluated whether pregnant women (gestational weeks 25–35) were at risk of exceeding a core body temperature of 39.0 °C during high-intensity running. We also investigated effects on skin temperature, fluid loss, and thermal sensation, comparing pregnant athletes to non-pregnant controls.

**Methods:**

In this comparative cross-sectional study, 30 elite and recreational athletes (pregnant n = 15) completed up to five high-intensity treadmill-intervals. Core and skin temperature were continuously measured. Body weight was utilized to calculate the amount of fluid loss.

**Results:**

Highest core body temperature were 38.76 °C and 39.56 °C in one pregnant and non-pregnant participant, respectively. Pregnant participants had lower core body temperatures (mean difference −0.47 °C, p ≤ 0.001) initially and a smaller increase (0.10 °C, p ≤ 0.003) during later intervals compared with the non-pregnant controls. Pregnant participants also showed a greater increase in skin temperature (4.08 ± 0.72 °C vs. 3.25 ± 0.86 °C, p = 0.008) and fluid loss (0.81 ± 0.19 L vs. 0.50 ± 0.12 L, p˂0.001).

**Conclusion:**

Physiological changes in pregnancy may enhance thermoregulation, indicating that high-intensity interval runs are unlikely to pose a risk of exceeding a core body temperature of 39 °C for pregnant athletes.

## 1 Introduction

Physical activity and exercise positively affect maternal, fetal, and neonatal health,^1^, ^2^, ^3^, ^4^ reducing risks of excessive gestational weight gain,^2^^,^^4^^,^^5^ preterm birth,^3^ gestational diabetes mellitus,^4^^,^^5^ preeclampsia,^4^^,^^6^ newborn complications^1^^,^^7^^,^^8^ and postpartum depression.^4^^,^^9^ All pregnant women without contraindications should engage in at least 150-min of moderate-intensity physical activity weekly.^10^, ^11^, ^12^, ^13^, ^14^ Athletes may undertake strenuous training routines involving high-intensity exercises (90 % of maximal heart rate),^10^^,^^15^, ^16^, ^17^ potentially impacting fetal well-being by redistribution of blood flow from the uterus to large muscle groups, possibly causing fetal bradycardia (<110 beats per minute (bpm)).^15^^,^^18^ However, no definitive upper limit for exercise intensity and duration during pregnancy has been established. Research in this field is limited^18^, ^19^, ^20^, ^21^ and inconsistent.^20^^,^^22^^,^^23^

High-intensity exercise increases body temperature,^24^ raising concerns regarding maternal heat stress and potential fetal impacts. The fetus, typically warmer than the mother, loses heat mainly through the uterine-placental circulation.^25^ Maternal heat stress can reduced placental blood flow, cause dehydration, and trigger inflammatory responses, potentially leading to preterm birth.^26^ Prolonged reduced uteroplacental blood flow can result in fetal growth restriction and low birth weight.^26^ Additionally, the first trimester's organogenesis stage is particularly sensitive to heat, potentially causing neural tube defects.^27^ The critical threshold is a maternal core body temperature exceeding 39.0 °C (°C).^15^^,^^25^ Most research is based on older animal and observational studies on fever-induced or passive heat stress hyperthermia.^28^, ^29^, ^30^, ^31^, ^32^, ^33^ There is a gap in understanding exercise-induced hyperthermia effects on healthy pregnant women, especially during high-intensity running. During pregnancy, it is presumed that thermoregulatory adaptions like lower core body temperature and increase sweat production, blood volume, skin blood flow, and skin temperature occur to protect the fetus.^34^, ^35^, ^36^, ^37^, ^38^, ^39^ These adaptations also include increased thermal heat capacity due to elevated body mass.^35^

Ravanelli et al.^40^ suggested that pregnant women can safely run for up to 35-min at 80–90 % of their estimated maximal heart rate, in environments below 25 °C. This conclusion is based on two small (n < 10) longitudinal studies from 1985 and 1987,^36^^,^^41^ with no control group. The few studies on high-intensity exercise in pregnancy highlight the need for further investigation. This study aimed to evaluate whether pregnant women (gestational week 25–35) exceed a core body temperature of 39.0 °C during high-intensity interval running and to compare the acute effects on skin temperature, fluid loss, and thermal sensation between pregnant elite and recreational athletes and non-pregnant controls.

## 2 Materials and methods

### 2.1 Design and participants

This comparative cross-sectional study was a part of the research project “*Strong Mama - the gender gap in sport medicine research: pregnancy, health, training, and motherhood in elite athletes*”.^42^ Data was collected between September 2022 and May 2023.

Due to limited research, we lacked sufficient data for power calculation and chose a sample of 30 Nordic Caucasian women, exceeding previous research participants counts.^36^^,^^41^ Fifteen pregnant elite and recreational athletes from *the Strong Mama project* consented to participate, and 15 non-pregnant controls were recruited via fitness clubs, social media, and students at the Norwegian School of Sport Sciences. Participants were informed about the study and test procedures, including temperature devices. Both groups were comparable (Table 1), each consisting of one elite athlete and 14 recreational athletes. An elite athlete was a member of any national team or other high-level representative team in any sport organized by a National Sports Federation. A recreational athlete regularly engaged in at least 240-min of endurance or strength training, including regular high-intensity sessions, for at least two years prior to their current pregnancy, or study enrolment for non-pregnant controls. Non-pregnant controls also had to use hormonal contraceptives to control for menstrual cycle influences. Background information collected included pre-pregnancy weight and training history. Most participants reported both endurance and strength training (pregnant n = 13 and non-pregnant n = 12), with the rest only reporting endurance training. Pregnant individuals with medical obstetric contraindications to exercise^43^ were excluded.

**Table 1:** Characteristics of pregnant and non-pregnant participants.

	Group			
	Pregnant		Non-pregnant	
Characteristics	n	Mean ± SD	n	Mean ± SD
Age (y)	15	31 ± 4	15	30 ± 6
Gestational age (weeks)	15	29.8 ± 2.9		–
BMI (kg/m^2^)	14	21.9 ± 1.7^a^^*,*^^b^	15	23.7 ± 2.1
Weight (kg)	15	72.2 ± 6.4	15	66.2 ± 5.7
GWG b	14	10.8 ± 2.4		–
Height (cm)	15	168.0 ± 5.7	15	167.4 ± 5.8
Exercise frequency (minutes/wk)^b^	14	342 ± 115.6	15	292 ± 50
Baseline rectal temp. (°C)	15	37.01 ± 0.21	15	37.51 ± 0.28
Baseline skin temp. (°C)	15	31.36 ± 0.79	15	30.20 ± 0.51
Estimated maximal heart rate^c^	15	197.0 ± 2.2	15	191.8 ± 7.6

BMI, body mass index = weight/(height)^2^; GWG, gestational weight gain; SD, Standard Deviation.

Significant differences between pregnant and non-pregnant participants, p < 0.05.

a Pre-pregnancy BMI (kg/m^2^).

b Self-reported data.

c Estimated maximal herat rate among the pregnant participants was calculated as earlier described by Mottola et al.(2006), and among the non-pregnant participants 220-age if the participants were unaware of their own maximal heart rate.

### 2.2 Measures

**Fig. 1:**
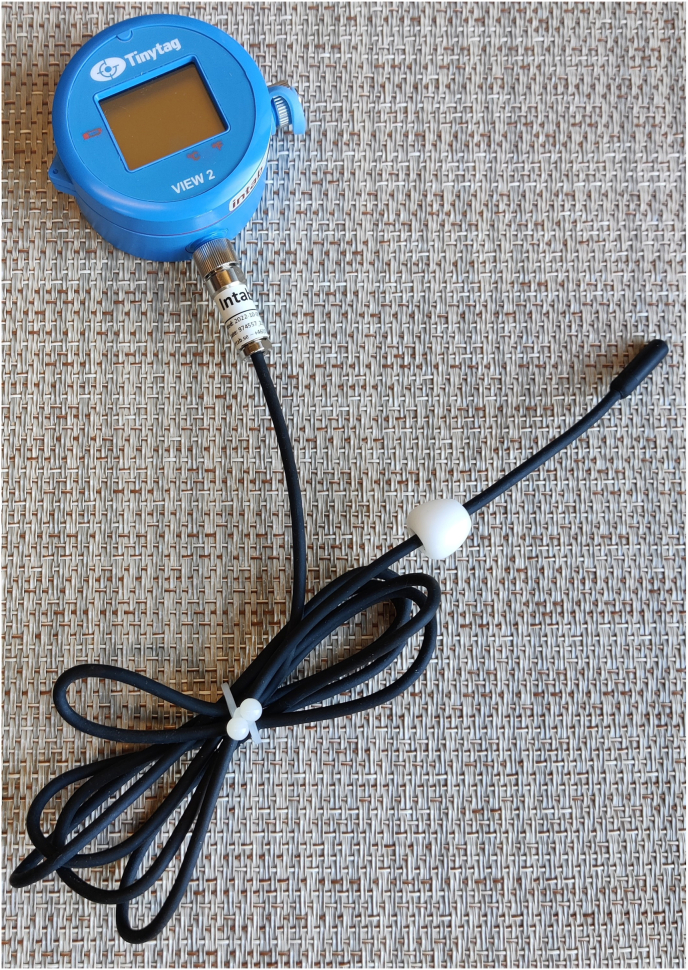
The rectal probe, Tinytag View2.

*Core body temperature:* Continuous core body temperature was measured using a rectal probe (PB-5015-1M5(3M), Gemini Data Loggers Ltd, West Sussex, UK), connected to a logging device (TinyTag® View 2 Temperature Logger, TV-4020, Gemini Data Loggers Ltd, West Sussex, UK). Participants self-inserted the rectal probe 10 cm past the anal sphincter following instructions from an exercise physiologist. A small stabilizer attached to the cable 10 cm from the sensor maintained the probe's depth (Fig. 1). Temperatures were recorded every second.

(Photo by Sofia Brevik-Persson)

*Skin temperature:* Skin temperature was measured using iButtons (Maxim Integrated Products Inc, iButtons DS1922L, San Jose, CA, USA). Specific measurement points were cleaned with alcohol and dried as needed. The iButton were attached with medical patches (Tegaderm I.V Advanced) at eight body sites according to ISO 9886 guidelines: (1) forehead, (2) right scapula, (3) left upper chest, (4) upper right arm, (5) lower left arm, (6) left hand, (7) right anterior thigh, and (8) left calf. Skin temperature was recorded at 1-mimute intervals.

*Fluid loss:* Body weight was measured pre- and post-exercise (Seca scale, model 877) in light clothing to the nearest 0.1 kg. Net fluid loss during the exercise session was calculated using the following equation:

Fluid loss (L) = body weight before exercise (kg) – body weight after exercise (kg) + fluid intake during exercise (L)

*Thermal sensation:* Thermal Sensation was measured at the end of each interval using a validated 8-point visual scale ranging from “Unbearably Cold” to “Neutral” to “Unbearably Hot”.^44^, ^45^, ^46^, ^47^, ^48^

### 2.3 Pilot test

Based on previous findings,^18^ the first six pregnant participants followed a modified protocol, discontinued interval running at an intensity of 17 on Borg's rating of perceived exhaustion (RPE) scale or 90 % of maximal heart rate. The gynecologist examined fetal health before and after each 5 min interval. Following successful pilot testing, the interval running was considered safe and subsequent participants (n = 9) followed a similar protocol.

### 2.4 Study protocol

Participants refrained from eating or drinking 2 h before the interval running and were contacted beforehand to ensure compliance. The treadmill protocol started with a 10-min warm-up of increasing intensity. Baseline temperatures were measured before the warm-up, with minute 0 marking the start of the first interval. Participants completed up to five 5-min treadmill-intervals (Woodway, Weil am Rhein, Germany) at target intensity (Borg RPE scale 17, 90 % of maximal heart rate). They initially determined their pace during the first interval to maintain the intensity. Speed was adjusted to maintain target intensity, with a constant 3° incline. A 4-min rest period followed each interval, during which pregnant participants had an ultrasound to ensure fetal safety, while no-pregnant controls sat during rest. Stopping criteria included abnormal fetal heart rate, low maternal oxygen saturation and participant discomfort. Abnormal fetal heart rate was defined as sustained fetal bradycardia (<110 bpm >3-min), fetal tachycardia (≥180 bpm during the investigation-pause between the treadmill intervals) and repeated decelerations. Participants could stop the protocol at any time. Temperature was recorded from 20-min before to 20-min after interval running, with a detailed time log documented each activity. All measurements were done in a laboratory at the Norwegian School of Sport Science, Oslo, by two exercise physiologists. Lab access was restricted to essential staff, with minimal opening of the door and human presence. Environmental conditions (20 °C–21 °C; relative humidity, 38 %–61 %) were monitored and recorded using a Kestrel 5400 Heat Stress Tracker (Pennsylvania, USA), to ensure consistency.

### 2.5 Missing data

During the intervals, one rectal probe showed a partial error measurement (−130 °C). Given the substantial deviation from normal core body temperature and the number of error data, it was considered appropriate to interpolate this data without significantly impacting the results. Temperature data between baseline and the initial interval were excluded for one pregnant participant due to improper insertion of the rectal probe. Additionally, one pregnant participant did not provide pre-pregnancy weight and training history responses.

### 2.6 Ethical approval

The study was approved by the Regional Committee for Medical and Health Research Ethics, Southern Norway, Oslo (REK 478976), and the Norwegian Social Science Service (Sikt 628051). All participants signed informed consent form, following the Helsinki Declaration. It was emphasized that participation was voluntary and that everyone who chose to participate could withdraw partially or fully from the project at any time without further explanation. Data was non-identifiable, and confidentiality was maintained in accordance with the law. The study was funded by The Norwegian Women's Public Health Association, with no compensation for participants. The IT department at the Norwegian School of Sports Sciences (NSSS) provides storage services, and Norwegian regulations require that all raw research data should be kept for at least five years after study completion.

### 2.7 Statistical analysis

Statistical analyses were performed using SPSS Statistical Software (IBM SPSS Statistics 29.0). Data were tested for normality by the Shapiro-Wilk Test, normality was assumed if p > 0.05. For differences between groups, a two‐sided independent sample *t*-test was used. Level of significance was set at p < 0.05. Data are presented as mean ± SD and range (min to max), along with reported differences between groups and corresponding 95 % confidence intervals (CI). Analyses were conducted on all participants, except one pregnant participant during the warm-up phase.

## 3 Results

Of the 15 pregnant participants, 10 were primiparous, while the remaining five had a history of uncomplicated pregnancies with a mean parity of 1.4. The mean gestational age among the pregnant participants was 29 weeks (range 25–35 weeks). Interval completion among pregnant participants was: five intervals (n = 4), four intervals (n = 4), three intervals (n = 5), and two intervals (n = 2). Discontinuation were due to participant preferences (n = 3), abnormal fetal heart rate during the investigation-pause between the treadmill intervals (≥180 bpm n = 1, <110 bpm n = 3), or exceeding 90 % of estimated maximal heart rate in the pilot group (n = 4). All non-pregnant participants (n = 15) completed five intervals. Both groups maintained high intensity during intervals two to five (Table 2).

**Table 2:** Mean intensity during each phase of the treadmill protocol in both groups.

Intensity	Group						*p*-value
	Pregnant			Non-pregnant			
	No.	% HR max	SD	No.	% HR max	SD	
Interval							
1st	15			15			
% HRmax		86	5		90	3	0.004
RPE		15	2		16	1	0.101
2nd	15			15			
% HRmax		89	4		93	2	0.001
RPE		16	2		17	0	0.109
3rd	13			15			
% HRmax		90	3		94	2	0.000
RPE		17	1		17	0	0.053
4th	8			15			
% HRmax		90	2		94	2	0.001
RPE		17	1		17	1	0.278
5th	4			15			
% HRmax		92	2		94	2	0.032
RPE		17	1		17	1	0.063

% HRmax, percent of estimated maximal heart rate; RPE, Borg's rating of perceived exhaustion.

SD, Standard Deviation.

### 3.1 Core body temperature

**Fig. 2:**
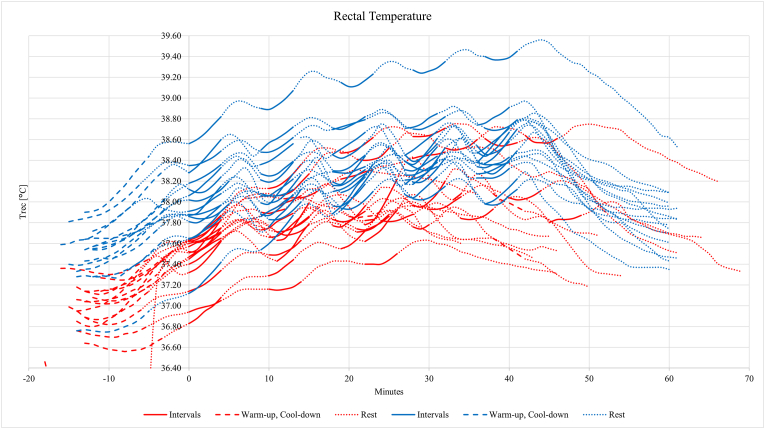
Rectal temperature changes during the treadmill protocol in pregnant (red) and non-pregnant (blue) participants. The first interval started at minute 0.

Compared with the controls, pregnant participants had a lower baseline core body temperature (37.01 ± 0.21 °C vs. 37.50 ± 0.28 °C, p < 0.001). This difference persisted during interval one (37.51 ± 0.25 °C vs. 38.04 ± 0.35 °C, p < 0.001), interval two (37.77 ± 0.27 °C vs. 38.19 ± 0.33 °C, p < 0.001), and interval three (37.92 ± 0.31 °C vs. 38.36 ± 0.33 °C, p = 0.001). Pregnant participants completing intervals four (n = 8) and five (n = 4) had a smaller core body temperature increases than controls (0.12 ± 0.05 °C vs. 0.22 ± 0.09 °C, p = 0.003) and (0.10 ± 0.01 °C vs. 0.19 ± 0.10 °C, p = 0.002, respectively). The core body temperature in pregnant participants increased by a mean of 1.19 °C (range 0.73–1.81 °C) above baseline, with the highest individual temperature at 38.76 °C (Table 3). Non-pregnant participants had a mean increase of 1.22 °C (range 0.59–2.06 °C), with the highest individual temperature at 39.56 °C. The highest individual temperatures in both groups occurred after interval five. Pregnant participants had a slower decline in core body temperature during the 20-min post-interval compared with controls, with individual variations shown in Fig. 2.

**Table 3:** Mean rectal temperature values recorded during each phase of the treadmill protocol for both groups.

Rectal temp.	Group						Group Difference	95 % CI		*p*-value
	Pregnant			Non-pregnant						
	No.	Mean	SD	No.	Mean	SD				
								Lower	Upper	
Baseline (°C)	15			15						
Avg		37.01	0.21		37.50	0.28	−0.49	−0.67	−0.31	<0.001
*Range [Min to Max]*		*36.56–37.37*			*36.76–37.91*					
Total exercise (°C)	14			15						
*Range [Min to Max]*		*36.64–38.76*			*36.74–39.56*					
Interval (°C)										
1st	15			15						
Avg		37.51	0.25		38.04	0.35	−0.53	−0.76	−0.30	<0.001
Min		37.40	0.26		37.94	0.35	−0.54	−0.77	−0.31	<0.001
Max		37.66	0.26		38.19	0.35	−0.53	−0.76	−0.30	<0.001
Inc		0.26	0.08		0.26	0.08	0.01	−0.05	0.07	0.747
*Range [Min to Max]*		*36.82–37.92*			*37.09–38.89*					
2nd	15			15						
Avg		37.77	0.27		38.19	0.33	−0.43	−0.66	−0.20	<0.001
Min		37.69	0.28		38.09	0.35	−0.40	−0.63	−0.16	0.002
Max		37.90	0.28		38.36	0.33	−0.46	−0.68	−0.23	<0.001
Inc		0.21	0.10		0.27	0.10	−0.06	−0.13	0.01	0.092
*Range [Min to Max]*		*37.15–38.36*			*37.52–39.13*					
3rd	13			15						
Avg		37.92	0.31		38.36	0.33	−0.44	−0.69	−0.19	0.001
Min		37.85	0.32		38.27	0.35	−0.42	−0.68	−0.16	0.003
Max		38.05	0.32		38.50	0.31	−0.46	−0.70	−0.21	<0.001
Inc		0.20	0.13		0.23	0.11	−0.03	−0.12	0.06	0.434
*Range [Min to Max]*		*37.39–38.63*			*37.86–39.28*					
4th	8			15						
Avg		38.16	0.33		38.44	0.30	−0.28	−0.57	0.01	0.055
Min		38.12	0.34		38.36	0.31	−0.24	−0.53	0.06	0.109
Max		38.25	0.32		38.58	0.29	−0.34	−0.61	−0.06	0.019
Inc		0.12	0.05		0.22	0.09	−0.10	−0.16	−0.04	0.003
*Range [Min to Max]*		*37.72–38.71*			*38.01–39.39*					
5th	4			15						
Avg		38.26	0.38		38.49	0.33	−0.22	−0.63	0.18	0.256
Min		38.23	0.38		38.42	0.34	−0.19	−0.06	0.22	0.335
Max		38.33	0.38		38.62	0.33	−0.29	−0.69	0.11	0.148
Inc		0.10	0.01		0.19	0.10	−0.10	−0.15	−0.04	0.002
*Range [Min to Max]*		*37.80–38.66*			*37.96–39.48*					

CI, Confidence Interval; Inc, increase temperature; SD, Standard Deviation; Total exercise, from warm-up to 20 min post-exercise.

Range [Min to Max]; Individually measured temperatures for all participants in each group.

A sample size below 15 is due to the occurrence of missing values due to discontinuation of the treadmill protocol (interval three to five) or measurement error during warmup (total exercise).

### 3.2 Skin temperature

**Fig. 3:**
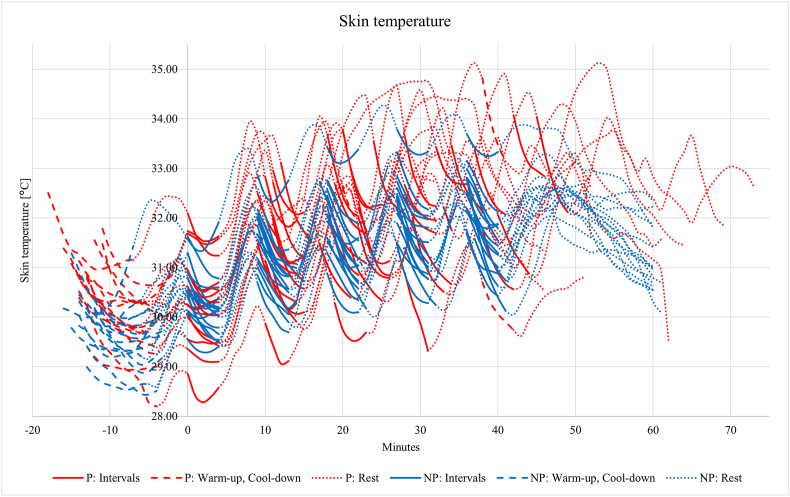
Skin temperature changes during the treadmill protocol in pregnant (red) and non-pregnant (blue) participants. The first interval started at minute 0.

Pregnant participants had significantly higher baseline skin temperature than non-pregnant controls (31.37 ± 0.79 °C vs. 30.20 ± 0.51 °C, p˂ 0.001). Their mean maximal skin temperature was also higher after the intervals (33.39 ± 0.94 °C vs. 32.44 ± 0.60 °C, p = 0.023) (Table 4). They experienced a greater increase in skin temperature from baseline to 20-min post-interval (4.08 ± 0.72 °C vs. 3.25 ± 0.86 °C, p = 0.008). The highest skin temperature among pregnant participants was 35.13 °C, measured five to 7 min after interval five, compared with 34.26 °C in non-pregnant controls. There were statistically insignificant skin temperature variations during each interval, with pregnant participants showing slightly higher values (Fig. 3).

**Table 4:** Mean skin temperature values recorded during each phase of the treadmill protocol for both groups.

Skin temp.	Group						Group Difference	95 % CI		*p*-value
	Pregnant			Non-pregnant						
	No.	Mean	SD	No.	Mean	SD				
								Lower	Upper	
Baseline (°C)	15			15						
		31.37	0.79		30.20	0.51	1.16	0.66	1.66	<0.001
*Range [Min to Max]*		*29.87–32.96*			*29.29–31.51*					
Total exercise (°C)										
Avg	15	31.62	0.76	15	31.28	0.54	0.34	−0.15	0.84	0.168
*Range [Min to Max]*		*28.20–35.13*			*28.44–34.26*					
Interval (°C)										
1st	15			15						
Avg		30.32	0.86		30.32	0.54	0.01	−0.53	0.55	0.976
Min		30.18	0.88		30.09	0.53	0.09	−0.45	0.64	0.728
Max		30.57	0.87		30.67	0.60	−0.10	−0.66	0.46	0.708
Inc		0.40	0.22		0.58	0.28	−0.18	−0.37	0.00	0.053
*Range [Min to Max]*		*28.28–32.09*			*29.29–31.92*					
2nd	15			15						
Avg		31.20	0.82		31.10	0.60	0.10	−0.44	0.64	0.705
Min		30.80	0.81		30.70	0.64	0.10	−0.45	0.64	0.712
Max		31.86	0.89		31.65	0.59	0.21	−0.35	0.78	0.446
Inc		1.61	1.06		0.95	0.25	0.12	−0.09	0.33	0.262
*Range [Min to Max]*		*29.06–32.08*			*29.70–32.86*					
3rd	13			15						
Avg		31.67	0.86		31.52	0.71	0.15	−0.46	0.76	0.621
Min		31.27	0.81		31.07	0.76	0.20	−0.41	0.81	0.508
Max		32.36	0.99		32.12	0.68	0.24	−0.42	0.89	0.464
Inc		1.09	0.37		1.05	0.43	0.04	−0.28	0.35	0.806
*Range [Min to Max]*		*29.54–33.79*			*30.10–33.43*					
4th	8			15						
Avg		31.95	0.95		31.75	0.72	0.20	−0.53	0.93	0.573
Min		31.56	1.08		31.34	0.81	0.22	−0.60	1.05	0.579
Max		32.53	0.97		32.36	0.67	0.18	−0.54	0.89	0.613
Inc		0.98	0.40		1.02	0.25	−0.05	−0.38	0.29	0.778
*Range [Min to Max]*		*29.32–33.63*			*30.24–33.28*					
5th	4			15						
Avg		32.55	0.81		31.81	0.67	0.73	−0.09	1.56	0.079
Min		31.98	0.81		31.35	0.78	0.63	−0.30	1.56	0.172
Max		33.39	0.94		32.44	0.60	0.95	0.15	1.75	0.023
Inc		1.41	0.35		1.10	0.43	0.31	−0.18	0.80	0.199
*Range [Min to Max]*		*30.88–34.08*			*30.13–33.25*					

CI, Confidence Interval; Inc, increase temperature; SD, Standard Deviation, Total exercise, from warm-up to 20 min post-exercise.

Range [Min to Max]; Individually measured temperatures for all participants in each group.

A sample size below 15 is due to the occurrence of missing values due to discontinuation of the treadmill protocol.

### 3.3 Fluid loss

After completion of five intervals, pregnant participants (n = 4) had higher overall fluid loss compared with non-pregnant controls (0.81 ± 0.19L vs. 0.50 ± 0.12L, p˂ 0.001). Including pregnant participants who completed four intervals (n = 8), the fluid loss remained higher (0.70 ± 0.19 vs. 0.50 ± 0.12L, p = 0.006). For relative sweat loss, pregnant participants completing all five intervals had an average sweat loss of 11.0 ml/kg compared with 7.5 ml/kg for non-pregnant controls (p = 0.003). Including those who completed four intervals, the average sweat loss was 9.4 ml/kg (p = 0.04). Fluid intake during the interval running was similar in both groups.

### 3.4 Thermal sensation

Thermal sensation ratings increased progressively with each interval for all participants, yet the pregnant participants generally rated lower on the thermal sensation (mean difference −0.8, p ≤ 0.04) than the non-pregnant controls (Table 5).

**Table 5:** Mean thermal sensation reported during each phase of the treadmill protocol for both groups.

Thermal sensation	Group						Group Difference	95 % CI		*p-*value
	Pregnant			Non-pregnant						
	n	Mean	SD	n	Mean	SD				
								Lower	Upper	
Interval										
1st	15	4.7	0.9	15	5.6	0.5	−0.9	−1.5	−0.9	0.003
2nd	15	5.2	0.7	15	6.0	0.6	−0.9	−1.4	−0.4	0.002
3rd	13	5.5	0.8	15	6.3	0.5	−0.8	−1.4	−0.3	0.002
4th	8	5.8	0.8	15	6.6	0.4	−0.8	−1.2	−0.2	0.007
5th	4	6.3	0.3	15	6.7	0.4	−0.5	−0.9	−0.0	0.038

Thermal sensation scale: 4.0 Neutral (Comfortable), 5.0 Warm, 6.0 Hot, 7.0 Very Hot.

CI, Confidence Interval; SD, Standard Deviation.

## 4 Discussion

We found that pregnant participants had a lower core body temperature during high-intensity interval running (17 on the Borg RPE scale, 90 % of maximal heart rate) compared with controls. Their temperatures consistently remained below the critical threshold of 39.0 °C, when training in environments around 20 °C. Pregnant participants also had higher peak skin temperatures, greater fluid loss, and reported lower thermal sensation than controls.

### 4.1 Core body temperature

Baseline core temperature was lower in the pregnant group compared to the non-pregnant group. According to previous research, core temperature during exercise appears to decrease progressively throughout pregnancy.^40^ Improved thermoregulation may result from increased skin circulation and higher skin temperature,^15^^,^^41^ facilitated by the natural decrease in vascular tone during pregnancy, enhancing heat dissipation during exercise.^15^^,^^24^ The results from the present study align with data from previous studies involving recreational runners and physically active women, where core body temperature never exceeded 39.0 °C.^35^^,^^36^^,^^40^^,^^41^^,^^45^ However, these findings were observed during steady-state running and at lower intensities. The use of percent of maximal heart rate makes the intensity unclear as the maximal heart rate is uncertain during pregnancy.^49^ In the present study, RPE was used, as recommended by ACOG,^10^ combined with the percent of maximal heart rate.

The previous studies have not shown exercise-induced increases in core body temperature exceeding 1.5 °C,^40^ with Clapp et al.^36^ observing increases ranging from 0.1 to 0.9 °C, Jones et al.^41^ reporting increases from 0.6 to 1.0 °C and Smallcombe t al.^45^ that report an increase <1.0 °C. However, the current study found that, although the mean increase remained below this threshold, individual increases did exceed it ranging from 0.73 to 1.81 °C. This suggests that high-intensity running at normal temperatures (20 °C) may elevate core body temperature more than lower-intensity exercises in warmer conditions (25–40 °C).^45^^,^^49^, ^50^, ^51^, ^52^ High-intensity exercise generates more heat than moderate-intensity exercise, even in elevated ambient temperatures,^45^ indicating that muscle activity impacts core body temperature more than heat exposure alone.^53^^,^^54^ Additionally, there was one case of tachycardia and three cases of bradycardia among the participants, all of whom had a slightly higher baseline core body temperature (ranging from 37.22 to 37.37 °C) compared to the average (37.01 ± 0.21 °C). However, whether this observation is more than a coincidence remains speculative and a larger sample size and more in-depth research into the acute responses of high-intensity exercise and abnormal fetal heart rate would be necessary to draw a conclusion. However, this was not specifically investigated in the present study and warrants further exploration.

### 4.2 Skin temperature

The increased skin temperature among pregnant participants, exceeding the peak values observed in controls, may be attributed to higher resting skin temperatures during pregnancy and alterations in blood flow distribution.^41^^,^^55^ Jones et al.^41^ reported that both exercise and resting skin temperatures increased with each trimester of pregnancy. Due to rapid changes in skin temperature, a standardized rest period before baseline evaluation is recommended,^56^ which our study did not include. Both groups skin temperature gradually increased after each interval, not following the core body temperature curve. Skin temperature decreased during running but increased after stopping, continuing to rise during rest, likely due to blood flow redistribution to working muscles. Smallcombe et al.^45^ measured skin temperature at four sites and found no significant difference between groups during moderate-intensity exercise, suggesting higher intensity exercise reveal more differences. In contrast, the present study measured skin temperature at eight sites to provide a more comprehensive assessment.

### 4.3 Fluid loss

Pregnant athletes experienced greater fluid loss than non-pregnant participants, likely due to a greater cardiac output, increased blood volume and reduced vascular resistance as well as a lower body temperature threshold for sweating and increased ventilation for heat dissipation.^25^^,^^35^^,^^57^ Few studies assessing sweat loss during pregnancy used different running protocols and intensities,^35^^,^^41^^,^^45^ complicating direct comparisons. However, previous studies suggest that physiological thermal protective changes occur throughout pregnancy.^25^^,^^35^^,^^41^ In a longitudinal study, Jones et al.^41^ observed that resting rectal temperature decreased as pregnancy progressed, but external work increased, resulting in consistent end-exercise sweat production. Clapp et al.^35^ followed 18 physically active pregnant women and found that the rectal temperature at which sweating began decreased by approximately 0.08 °C per month of gestation, indicating improved heat loss responses as pregnancy advanced. Smallcombe et al.^45^ reported no differences in sweat loss between groups, and with their pregnant participants losing 0.54L less compared to the pregnant participants in the present study under restricted fluid intake. However, in the same study the exercise intensity (75 % of maximal heart rate) might have been too low despite the high ambient temperature (32 °C). In the present study, participants were assumed to be well-hydrated, as fluid intake was not restricted. Dehydration could affect sweat production in response to rising core body temperature.^58^^,^^59^ The potential for sweat to be trapped in the lightweight clothing was deemed negligible and not adjusted for.^60^

### 4.4 Thermal sensation

Despite elevated skin temperatures, pregnant participants reported greater thermal comfort during high-intensity interval running than non-pregnant controls. This contrasts with Smallcombe et al.,^45^ who found pregnant participants felt warmer than non-pregnant controls. Our participants were athletes, likely more accustomed to high-intensity exercise, which may explain their lower discomfort ratings. Smallcombe et al.^45^ did not report participants' exercise familiarity. A thermal comfort scale may not reliably reflect self-perceived core body temperature in pregnant athletes exercising at normal room temperatures.

### 4.5 Practical implications

Our findings suggest that pregnant athletes in late second to early third trimester can perform 5-min high-intensity interval running with 4-min rest periods without risk of exceeding a core body temperature of 39 °C. Pregnant women should stay well-hydrated before, during, and after exercise to support thermoregulation.^43^ Outdoor exercise benefits from wind aiding heat loss, while indoor exercise may be better in hot, humid conditions. Post-exercise, core body temperature can remain elevated, so further activity may increase it.^61^ Factors like gestational age, exercise timing, and individual heart rate should be considered, as some pregnant women may be more vulnerable to heat stress.

### 4.6 Perceptual responses of running with a rectal probe

Participants reported similar perceptual responses to running with a rectal probe. They acknowledged discomfort during sensor insertion and removal but found the probe less noticeable while running.

### 4.7 Strength and limitations

Our study on pregnant elite and recreational athletes in late second to early third trimester examined the acute effects of high-intensity interval running on body temperature and thermoregulation. Comparing these athletes with non-pregnant controls allow us to assess the impact of pregnancy on thermoregulation during high-intensity exercise. We measured core and skin temperature, fluid loss, and thermal sensation to provide a comprehensive view of high-intensity exercise effects on pregnant women. Precise and continuous measurements were obtained using a rectal probe and iButtons®, with body weight used to calculate fluid loss. The rectal probe, considered the gold standard for core temperature measurement, was equipped with a stabilizer to prevent slippage. Live temperature displays ensured accurate data collection, and hormonal contraceptive use among non-pregnant participants controlled for menstrual cycle variations and the effect on the core body temperature.^62^ All intervals were under controlled conditions with a gynecologist monitoring fetal parameters for safety.

Fewer pregnant participants (n = 4) completed all five intervals compared with controls (n = 15), affecting statistical analysis reliability. Focusing on a single session limit understanding of long-term exercise effects on temperature regulation. Circadian variations in physiological responses may have influenced the results, as core body temperature fluctuates during the day.^56^ The consistent 4-min pause during intervals may not reflect real-life practice, potentially underestimating core body temperature. Conducting the protocol at normal room temperatures may limit applicability to warmer climates.

### 4.8 Future research

There may be variations in maternal temperature response related to intense physical exercise earlier in pregnancy, and research is needed to investigate these differences. Considering that athletes often train multiple times a day and core body temperature can stay elevated for up to 24 h post-exercise,^61^ exploring the impact of subsequent activities on core body temperature is valuable. Future research could also aim to implement continuous fetal heart rate monitoring (cardiotocography) during exercise.

Finally, the research team actively followed all pregnant participants in the present study until delivery. Each pregnancy resulted in the birth of a healthy term infant with no adverse outcomes or complications reported.

## 5 Conclusions

Our study found that core body temperature increased during high-intensity interval running among pregnant elite and recreational athletes but remained below 39.0 °C. Compared with non-pregnant athletes, pregnant participants had a smaller increase in core body temperature, lower thermal sensation ratings, but higher skin temperature and greater fluid loss. Our findings suggest possible thermoregulatory improvements during pregnancy and high-intensity exercise.

## Author contributions

LAHH is the main supervisor and initiative taker for *the Strong Mama project* and has served as mentor for the Cool Mama project together with CG. SBP has outlined the present manuscript and been responsible for data collection together with EMD. JM has provided his academic expertise in the field of temperature, as well contributed to data interpretation, and provided critical feedback together with LAHH, CG and EMD. As a medical responsible within *the Strong Mama project*, BS has played a vital role, leveraging her expertise to ensure the well-being and safety of all participants involved.

All authors reviewed and approved the final manuscript, and the publication has been approved by the responsible authorities at the institution where the work has been carried out.

## Funding

This project is funded by the Norwegian women's Public Health Association.

## Data availability statement

The datasets used and analyzed in this study are available from the first author on reasonable request.

## Declaration of competing interest

The authors declare there are no conflicts of interest. The source of funding did not have any role in the study's design, conduct, data collection, management, analysis, or interpretation, nor did it influence the manuscript's preparation, review, approval, or the decision to submit it for publication.
